# In situ production of titanium dioxide nanoparticles in molten salt phase for thermal energy storage and heat-transfer fluid applications

**DOI:** 10.1007/s11051-016-3460-8

**Published:** 2016-06-07

**Authors:** Mathieu Lasfargues, Andrew Bell, Yulong Ding

**Affiliations:** School of Chemical and Process Engineering, University of Leeds, Leeds, LS2 9JT UK; School of Chemical Engineering, University of Birmingham, Birmingham, B15 2TT UK

**Keywords:** Molten salt, Nanoparticles, Specific heat capacity, Nitrate salt, Heat-transfer fluid, Nanoparticle production

## Abstract

In this study, TiO_2_ nanoparticles (average particle size 16 nm) were successfully produced in molten salt phase and were showed to significantly enhance the specific heat capacity of a binary eutectic mixture of sodium and potassium nitrate (60/40) by 5.4 % at 390 °C and 7.5 % at 445 °C for 3.0 wt% of precursors used. The objective of this research was to develop a cost-effective alternate method of production which is potentially scalable, as current techniques utilized are not economically viable for large quantities. Enhancing the specific heat capacity of molten salt would promote more competitive pricing for electricity production by concentrating solar power plant. Here, a simple precursor (TiOSO_4_) was added to a binary eutectic mixture of potassium and sodium nitrate, heated to 450 °C, and cooled to witness the production of nanoparticles.

## Introduction

**Fig. 1:**
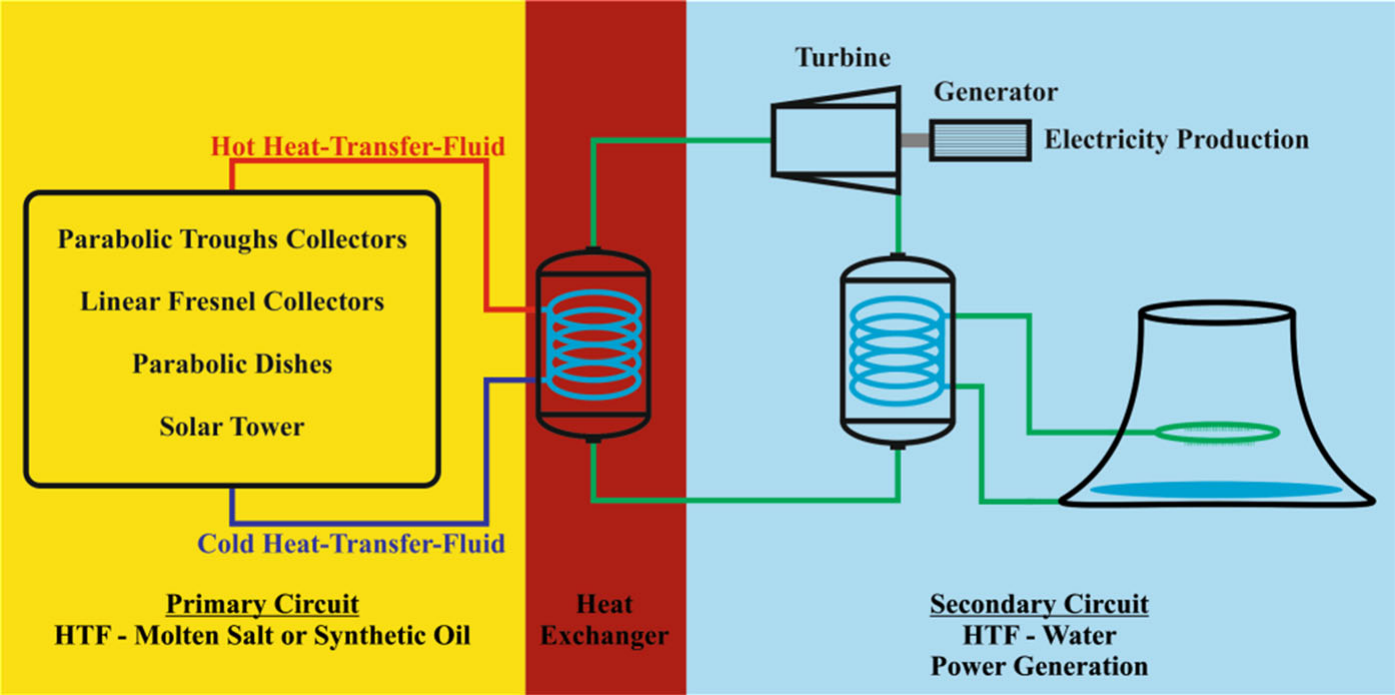
Schematic diagram of CSP plant—currently most plant utilizing molten salt run at a maximum of ≈560 °C with a minimum of 290 °C, whilst synthetic oil, such as VP1 Therminol, has a temperature range of 13–395 °C. Energy storage either direct or indirect is a viable option for this technology and is implemented in many CSP plants (Price et al. 2002; Medrano et al. 2010)

Concentrated Solar Power (CSP) technology operates through the collection and concentration of solar radiation utilizing the long-wave region of its spectrum as a source of energy (Gil et al. 2010; Medrano et al. 2010). The latter is stored and transported by the heat-transfer fluid (HTF) to the heat exchanger to produced steam and power turbine generating electricity in the process (Fig. 1). Currently, synthetic oils are commonly used in the primary loop system, but their low thermal stability since they decompose at operating temperatures exceeding 400 °C as well as toxicity, high purchasing cost, and high vapour pressure is pushing industrials and academics toward alternative materials to solve these challenges (Cavallaro 2010). Molten salt (Solar Salt) is currently seen as a viable option due to its low toxicity, cost and vapour pressure, as well as good thermal stability, since they decompose at operating temperatures exceeding 600 °C. However, their high freezing point and low thermo-physical properties are a disadvantage of using this material which might be offset by higher thermodynamic efficiency attained when running a CSP plant at 560 °C rather than 395 °C.

Current research is looking at enhancing the specific heat capacity through the addition of nanoparticles within the molten salt mixtures. The presence of nanoparticles into liquid (nanofluids) has been extensively studied and stems from the work of Choi and Eastman (1995), whereby anomalous enhancement of thermal conductivity was reported when nanoparticles (Al_2_O_3_, Au, Ag, Cu, TiO_2_, CuO, Carbon Nanotube, ZnO, Graphite, Graphene, SiC, etc.) (1–100 nm) were suspended into various liquid phase (Pumped oil, ethylene glycol, water, toluene, etc.) (Lee et al. 1999; Wang et al. 1999; Xuan and Li 2000; Eastman et al. 2001; Tsai et al. 2004; Yang et al. 2005; Kang et al. 2006; He et al. 2007; Zhang et al. 2007; Yu et al. 2009; Moosavi et al. 2010; Paul et al. 2010; Lee et al. 2011; Sharma et al. 2011; Yu et al. 2011). With thousands of papers studying these anomalous enhancements, the various factors (size of particle, shape, surface properties, concentration, and interaction with base fluid) encompassing this rise has made it difficult to model accurately (Xuan and Li 2000; Das et al. 2003, 2006; Lee et al. 2006; Prasher et al. 2006; Ding et al. 2007; Trisaksri and Wongwises 2007; Yang and Liu 2010; Yu and Xie 2012). This said, recent theory and understanding point to the effective medium theory by Maxwell as a good model for prediction of thermal conductivity (Keblinski et al. 2008; Buongiorno and Venerus 2009).

Whilst thermal conductivity of nanofluids has been extensively studied at low temperature for non-ionic fluids, very little has been published on high-temperature ionic fluid in comparison. Much like, non-ionic fluid, the preparation and suspension of nanoparticles into molten salt is going to be affected by the methodology employed in its making.

**Fig. 2:**
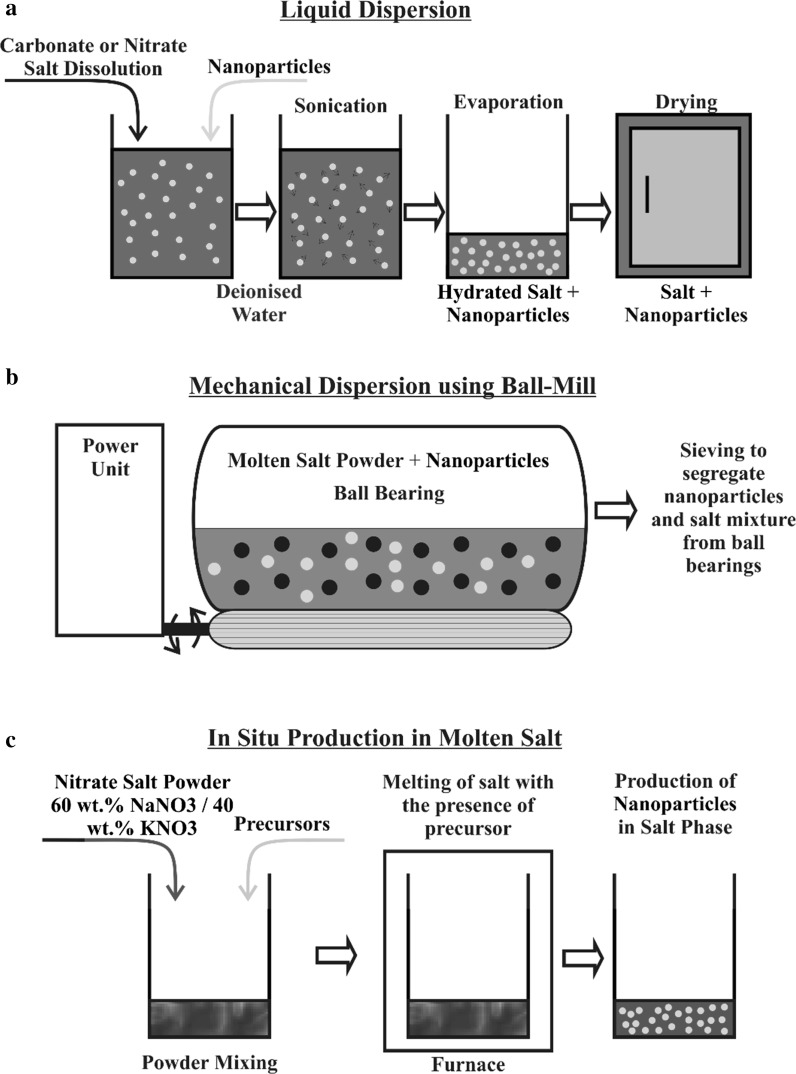
Various methodologies that can be employed in the making of molten salt nanofluids. Whilst liquid dispersion (**a**) is the most used technique (Shin and Banerjee 2010, 2011a, b; Jo et al. 2012; Chieruzzi et al. 2013; Dudda and Shin 2013; Lu and Huang 2013; Tiznobaik and Shin 2013a, b; Andreu-Cabedo et al. 2014; Jo and Banerjee 2014; Chieruzzi et al. 2015; Tao et al. 2015), mechanical dispersion (**b**) can also be used (Lasfargues et al. 2015) as well as In Situ production (**c**) which has not been implemented in any research and will be looked at in this paper

The most utilized technique in the production of molten salt nanofluids is the dispersion/dissolution in water coupled with sonication and drying which stems from the works of Shin (2011) and Jung (2012) thesis dissertations and also led to a patent application on a technique of production of in situ nanoparticles and nanofins (Shin and Banerjee 2010; Shin 2011; Shin and Banerjee 2011a, b; Jo et al. 2012; Jung 2012; Chieruzzi et al. 2013; Dudda and Shin 2013; Khodadadi et al. 2013; Lu and Huang 2013; Tiznobaik and Shin 2013a, b; Andreu-Cabedo et al. 2014; Banerjee and Jo 2014; Jo and Banerjee 2014; Chieruzzi et al. 2015; Tao et al. 2015) (Fig. 2a). Whilst this technique scatters the nanoparticles and potentially destroy aggregates that have formed, the evaporation of water might still promote their formation, as the volume gets reduced and the concentration of nanoparticle rises which is what is been observed by Shin and Banerjee (2013) in Fig. 1 where a fine and coarse structure is clearly visible in the petri-dish (Shin and Banerjee 2013). These changes in the spatial arrangement of the nanoparticles led to variation in specific heat capacity, whereby coarse structure (aggregated nanoparticles) showed no enhancement, whilst fine/dispersed nanoparticles displayed increases of 118–124 % compared to the base salt (Shin and Banerjee 2013). The theory behind these enhancements are due to the production of compressed layer (semi-solid layer of ions) around the formed nanostructure which are described by Shin and Tiznobaik (2014) as fractals (Shin and Tiznobaik 2014; Jo and Banerjee 2015). It has also been hypothesised that the surface phonon which are usually negligible might, due the large surface area-to-volume ratio, play a vital role in this increase in specific heat capacity.

Mechanical dispersion (Fig. 2b) is an alternate approach and could potentially be leveraged for scale-up synthesis. However, more research needs to be implemented on its effect and how to control the homogeneity of the mix. The size and type of bearing used, as well as the amount of time the mixture is homogenised and its speed is going to impact the uniformity of the mix in different ways. In this paper, we explore the in situ synthesis of titanium oxide nanoparticles in the molten salt phase [60 wt% Sodium nitrate (NaNO_3_) and 40 wt% Potassium Nitrate (KNO_3_) eutectic mix] using a precursor (Fig. 2c), such as titanium (IV) oxysulphate (TiOSO_4_), and how this might affect the specific heat capacity (Cp) of the mixture. The precursor was chosen because of TiO_2_ many applications throughout different industries (Dye in paint, paper, plastics, medicine, food; Sunscreen blocker; Photo-catalyst; Batteries) (Zhu et al. 2012).

## Methodology

### Sample production

Anhydrous NaNO_3_ (FISHER, UK) (>99 % Pure) and KNO_3_ (SIGMA-ALDRICH, UK) (>99 % Pure) as well as TiOSO_4_ (SIGMA-ALDRICH, UK) were purchased for these tests. The salt and precursors were mixed using a pestle and mortar with the following ratio (Table 1).

**Table 1:** Amount of each component used in the production of molten salt nanofluids (1.0 wt% analysed with SEM, EDX, BSE, and TEM, whilst Cp was carried out for all of the precursors made)

Percentage of precursor (%)	Total weight (g)	NaNO_3_ (60 wt%) (g)	KNO_3_ (40 wt%) (g)	Precursor (TiOSO_4_) (g)
1.00	12.50	7.42	4.95	0.13
2.00	12.50	7.35	4.90	0.25
3.00	12.50	7.27	4.85	0.38

**Fig. 3:**
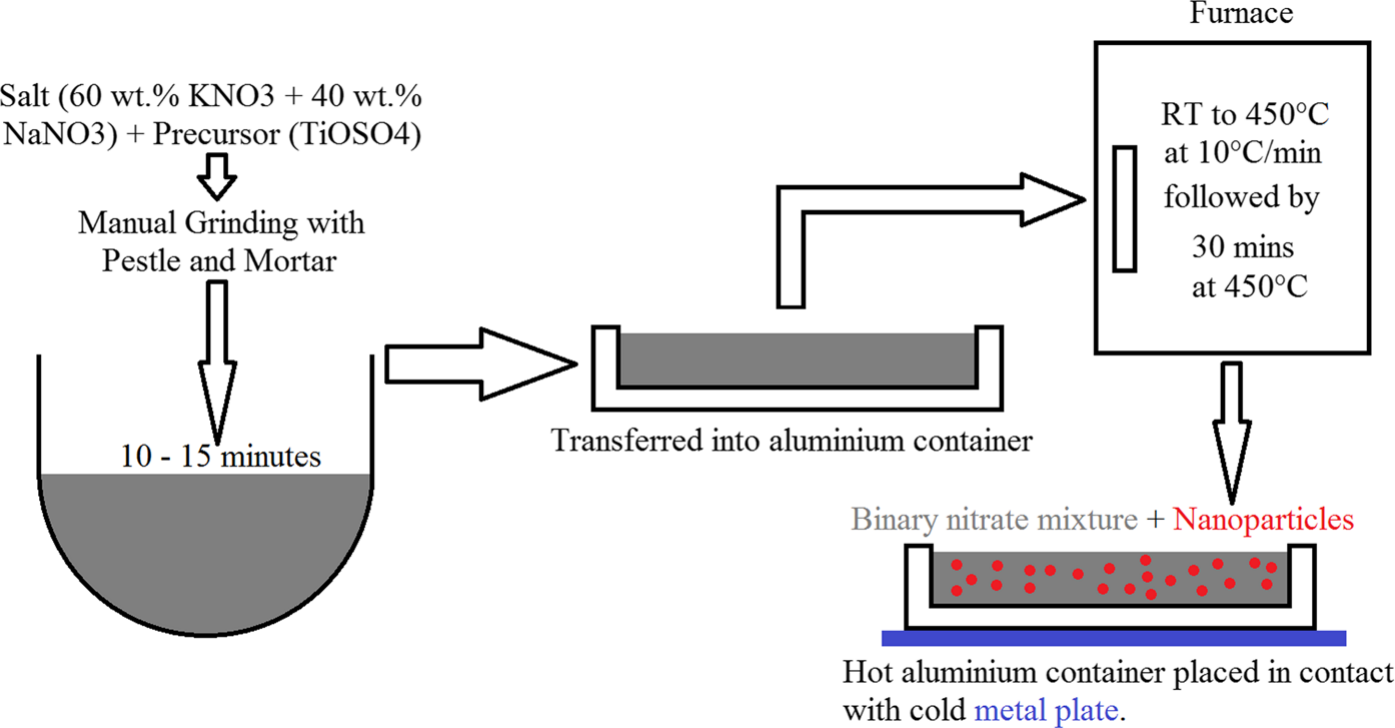
Flow diagram of how the process for the production of in situ nanoparticles in molten salt phase is carried out

After grinding the mixture for 10–15 min manually, the latter was transferred to an aluminium crucible and inserted in a furnace (Carbolite, 1100, OAF 11/2, UK). The thermal program used for the sample production was a ramp up from room temperature to 450 °C at 10 °C/min with an isothermal of 30 min at 450 °C. Once finished, the crucible was removed and left to cool on a stainless steel sheet of metal (Fig. 3). This promoted a rapid temperature drop and crystallisation of the salt.

Once cooled the samples were analysed using scanning electron microscope (SEM) coupled with energy dispersive X-ray (EDX) for elemental analysis as well as backscattered electron (BSE) and transmission electron microscopy (TEM). Finally, utilizing thermo-gravimetric analyser combined with differential scanning calorimeter (DSC) sensor as well as a stand-alone DSC, the production of nanoparticles was analysed and the specific heat capacity (Cp) was measured.

### SEM, BSE, and EDX

Morphological and elemental analysis of the samples was carried out using a field emission SEM (Hitachi SU8230, Berkshire, UK). Samples were mounted on 12 mm aluminium SEM stub and coated with a 3 nm mixture of platinum/palladium using an 80/20 ratio, via a high-resolution sputter coater.

### TEM

Further analysis of the nanoparticles themselves was carried out using transmission electron microscopy (FEI TECNAI F20, Cambridge, UK). The samples were produced by dissolving the salt containing the produced nanoparticles into deionised water and sonicating the mixture for a couple of hours. Then, it was centrifuged, the supernatant was drained, and more deionised water was added with a further round of sonication to re-suspend the nanoparticles. This was repeated a number of times to remove the nitrate salt. Finally, using a 10 µl Gilson pipette, a small sample was withdrawn, applied onto a 3 mm copper grid and left to dry at room temperature before analysis.

### DSC

The specific heat capacity was measured using a heat-flux DSC with automated sampler (Mettler Toledo, DSC-1 700, Leicester, UK). The samples used were first grinded in a pestle and mortar and inserted inside 30 µl platinum crucibles (Mettler Toledo, UK) with amounts varying between 30,000 and 35,000 mg. The samples were repeatedly melted and frozen several times (Table 2) before testing the Cp values. The thermal cycle employed was an isothermal of 5 min at 250 °C followed by a rapid ramp up to 450 °C at a rate of 40 °C/min with a final isothermal of 5 min at 450 °C. A sapphire standard (24.055 mg) was used as a standard to calculate the Cp.

**Table 2:** Melting and enthalpy of the different samples produced

	Melting point (°C)	SD	Enthalpy of fusion (J/g)	SD
Pure salt	220.37	0.45	−116.02	0.43
1.0 wt% TiOSO_4_	217.67	0.18	−115.27	0.14
2.0 wt% TiOSO_4_	217.46	0.03	−113.59	0.49
3.0 wt% TiOSO_4_	217.50	0.05	−110.18	0.53

**Fig. 4:**
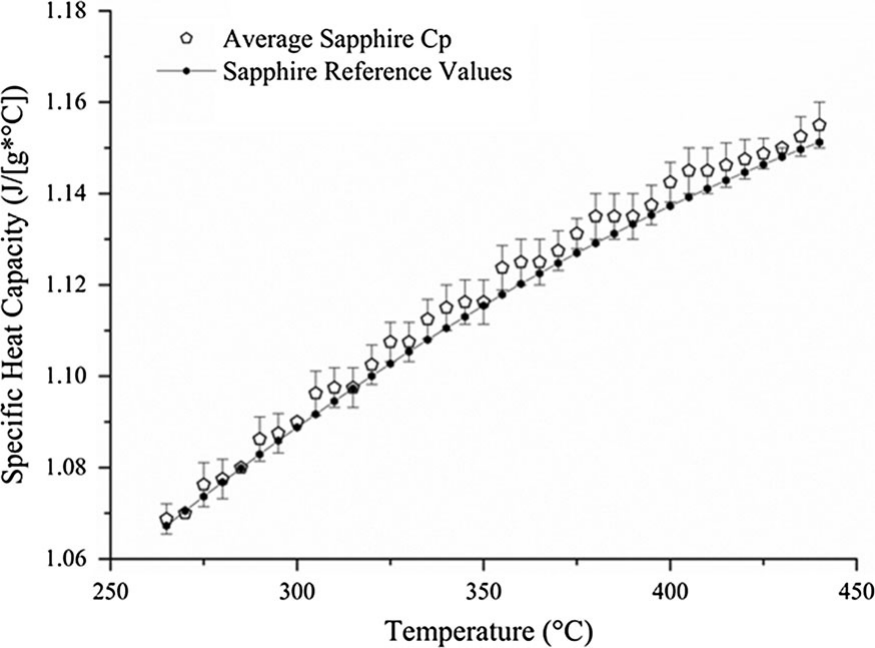
Comparison between experimental and reference values of sapphire for specific heat capacity (Tiznobaik and Shin 2013b)

As always the specific heat capacity of sapphire was checked to ensure the validity of this methodology. With results within ±0.55 % of the reference sapphire value, the process was considered precise and accurate enough to carry on the tests (Fig. 4).

### TGA/DSC

The production of nanoparticles in molten salt phase was followed using a TGA/DSC (Mettler Toledo, TGA/DSC, LF-1100, Leicester, UK). The sample was a combination of sodium and potassium nitrate (60/40) mixture with titanium oxysulphate. To obtain large enough peaks to analyse and as the aim of this test was just to observe how the decomposition reaction took place, ≈27.000 mg of salt (60 wt% NaNO_3_+ 40 wt% KNO_3_) was mixed with ≈3.000 mg of TiOSO_4_ and inserted inside a platinum crucible (40 µl). The thermal sample employed was an isothermal of 30 min at 50 °C followed by a ramp up to 450 °C at 10 °C/min with another isothermal at 450 °C for 30 min.

## Results and discussion

**Fig. 5:**
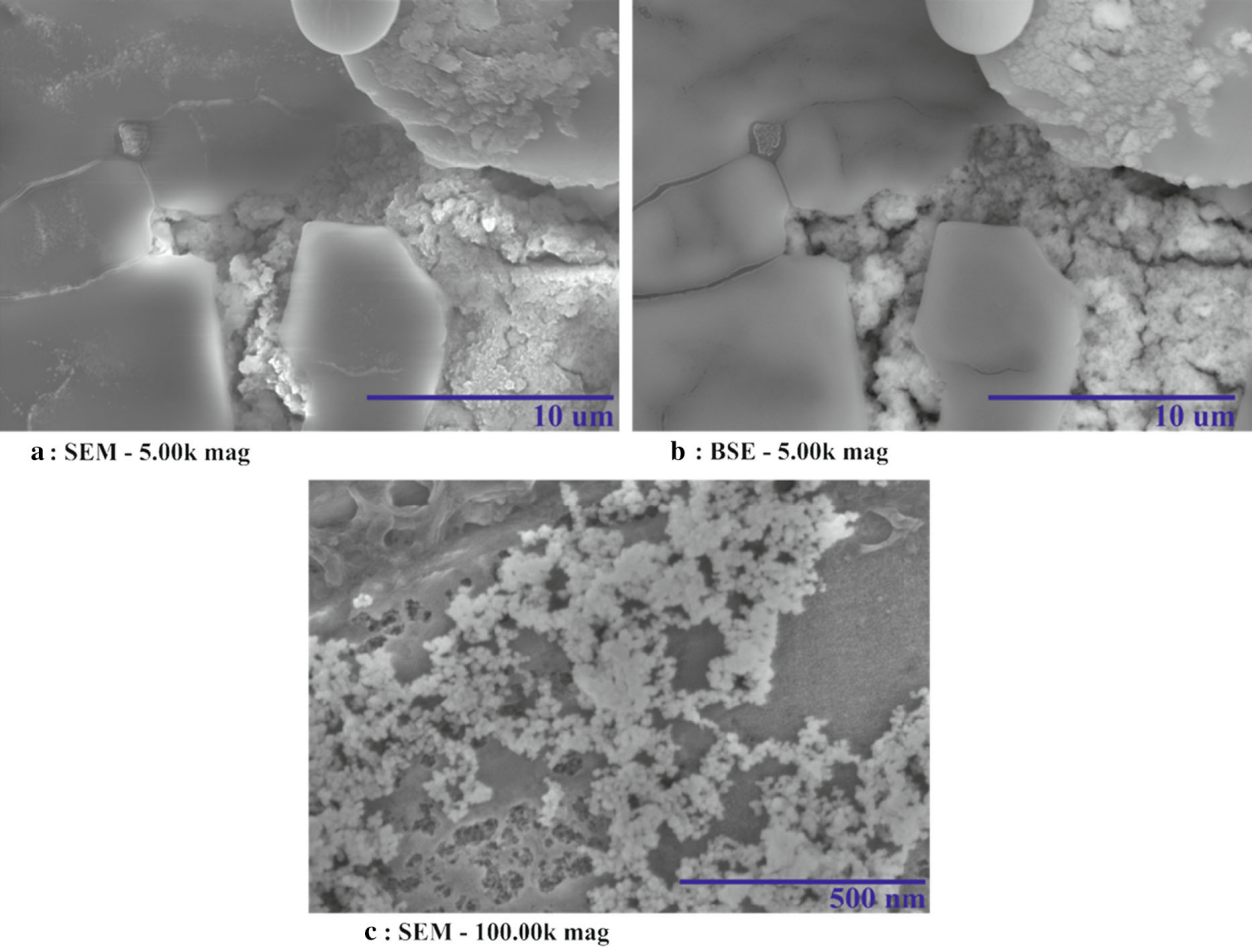
**a**–**c** Images taken using either SEM or BSE of nanoparticle production using 1.0 wt% TiOSO_4_ precursor in molten salt phase (60 wt% NaNO_3_+ 40 wt% KNO_3_)

**Fig. 6:**
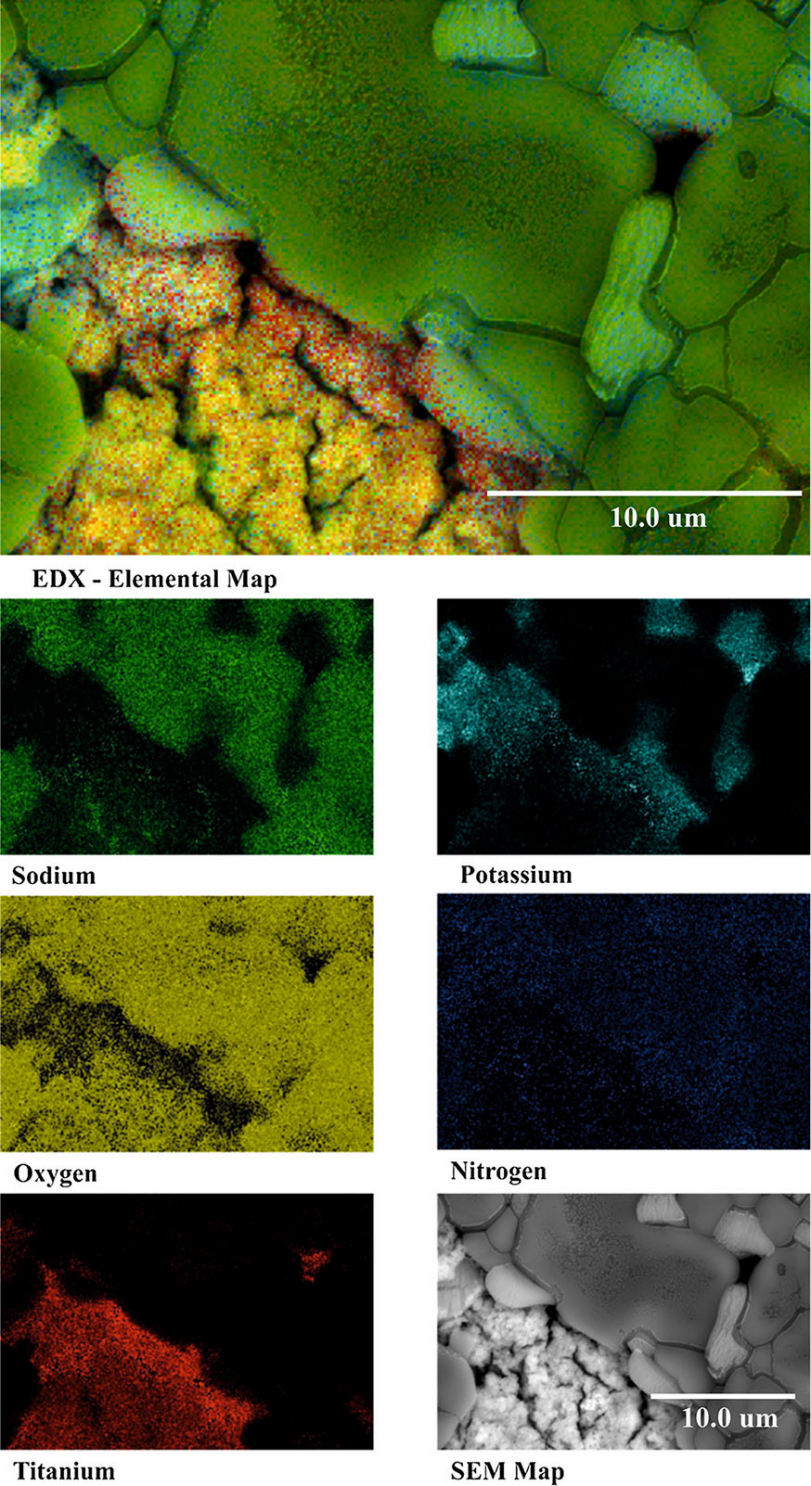
EDX analysis—the top picture shows an overlay of all the elements present in the sample followed by several maps showing the position of each individual element (Na–*green*, K–Cyan, O–*yellow*, N–*dark blue*, and Ti–*red*). The *bottom right* picture is an SEM map of the area scanned. (Color figure online)

The SEM analysis of the lowest concentration of TiOSO_4_ precursors (1.0 wt%) displayed what seemed to be aggregated nanoparticles (Fig. 5a). This is partially corroborated by backscattered electron analysis which clearly highlight a density change between the molten salt which appear dark grey and the nanoparticles which have a lighter colour (Fig. 5b). Furthermore, the BSE picture clearly showed that the adsorption of nanoparticles onto the molten salt surface (Fig. 5b: Top right) (Lu and Huang 2013; Chieruzzi et al. 2015) and a magnification of 100,000 times (Fig. 5c) seem to show that the nanoparticle in question are relatively homogenous in term of size. The elemental mapping utilizing the EDX (Fig. 6) revealed the presence of titanium and oxygen which looked to be superimposed implying the production of TiO_2_. This would make sense as the thermal decomposition of this precursor is as follow:

$${\text{TiOSO}}_{{4({\text{g}})}} \to {\text{TiO}}_{{2({\text{s}})}} + {\text{SO}}_{{2({\text{g}})}} + \raise.5ex\hbox{$\scriptstyle 1$}\kern-.1em/ \kern-.15em\lower.25ex\hbox{$\scriptstyle 2$} {\text{O}}_{2} \left( {{\text{Johnsson et\, al}}. \,{ 1997}} \right).$$

In this molten state, the ions would not take part in the reaction and the completion of the reaction could be partly indicated by the presence or absence of sulphur which was definitely not picked up by the EDX as a major component of this sample. Another interesting revelation provided by the EDX analysis was the packing of sodium and potassium atoms in relation to titanium. From Fig. 6, it can be seen that the potassium atoms are able to come into closer contact to the titanium than sodium. This could probably be explained by the fact that the larger ions are a better fit requiring less packing energy, thereby reducing entropy (better adhesion properties between the two materials). The EDX also showed that the potassium atoms are segregated from the sodium with very faint overlapping seen when close to the nanoparticle’s surface. This has been demonstrated by Jo and Banerjee (2014), in their molecular dynamic model which describe these chemical potential cause by enhanced adhesion properties between potassium and graphite in the compressed layers as an element which might be part of the increased storage mechanism seen in molten salt nanofluids (Jo and Banerjee 2014).

**Fig. 7:**
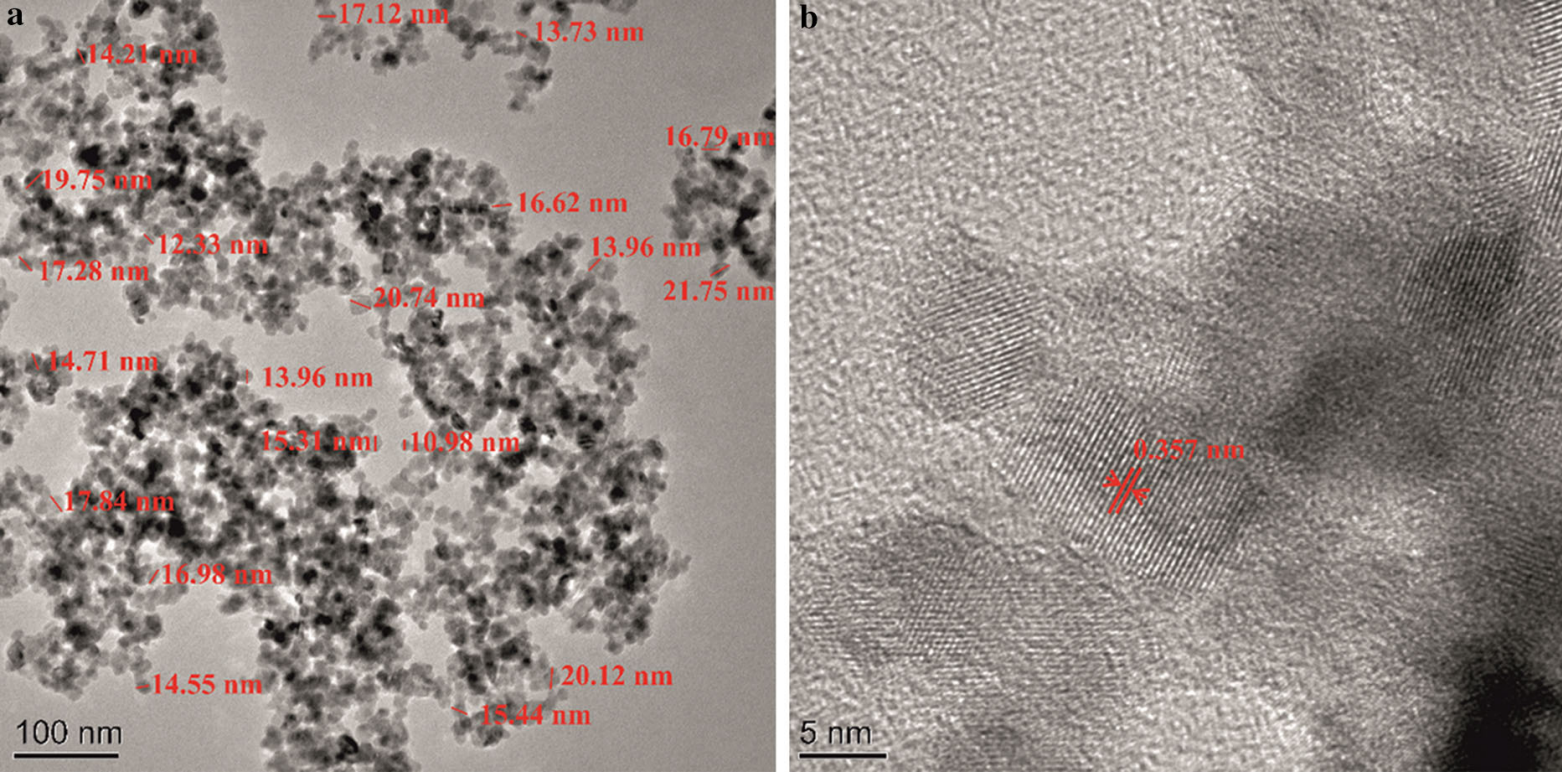
**a**, **b** TEM pictures taken at different magnifications for nanoparticles produced in molten salt phase (60 wt% NaNO_3_+ 40 wt% KNO_3_) using 1.0 wt% TiOSO_4_ precursor

Analysis of the TEM images showed that the nanoparticles produced were homogenous in nature with an average diameter estimated at 16.35 nm ± 2.80 (Fig. 7a). The TEM diffraction pattern confirmed that anatase was produced (Fig. 7b). The lattice distance came to an average of 0.357 nm (Fig. 7b) which is in good agreement with the previous studies for the (101) plane of the anatase phase (Xie et al. 2009; Dai et al. 2010). The production of nanoparticles in molten salt state is definitely achievable, although it leads to the production of aggregates which would have to be broken down through the use of sonication at high temperature. Furthermore, the time, amount of precursors, maximum temperature, and production method used in the nucleation process are likely to affect the size of the nanoparticles.

**Fig. 8:**
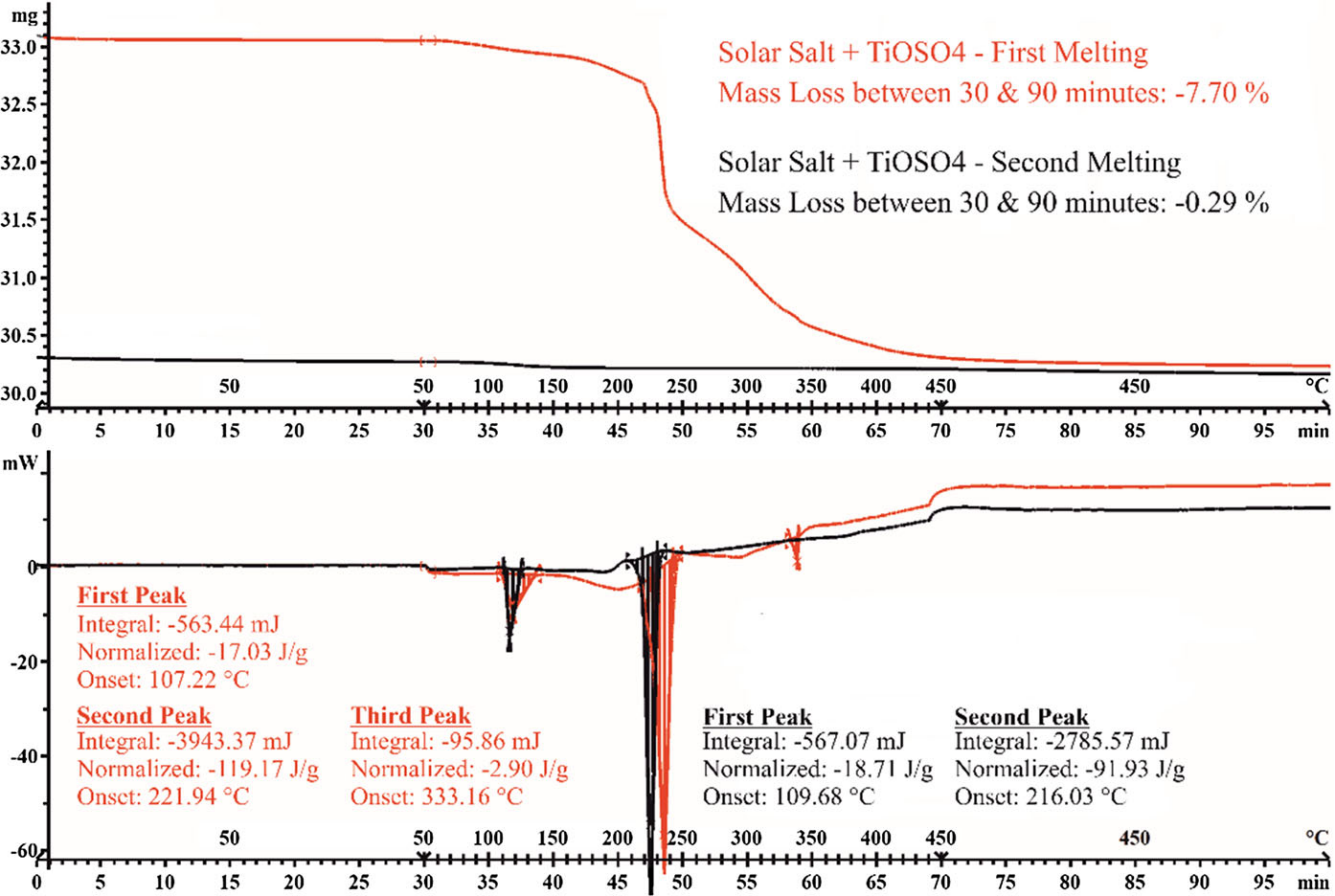
TGA–DSC curves of TiOSO_4_ precursors mixed with solar salt for nanoparticle production using the same thermal cycle to that of the furnace except for the original isothermal at 50 °C which is used to allow the balance to stabilise

From the TGA-DSC analysis (Fig. 8), it can be seen that the first melting cycle leads to the production of three endothermic peaks with an onset of 107.22, 221.94, and 333.16 °C, whilst the second melting cycle of the same sample only yield two at temperature of 109.68 and 216.03 °C. The first endothermic peak is likely to be water content/moisture, and both curves display this peak combined with mass loss. The second endothermic peak will be that of the salt melting which given the temperature at which it occurs correspond to a eutectic binary mixture of sodium and potassium nitrate (60/40). The third endothermic peak is only present in the first melting cycle and absent in the second. It is probably caused by the production of titanium sulphate (Ti(SO_4_)2) which has a boiling point of 330 °C. It is also important to notice that the sharpest drop in sample weight occurs right after the melting of the salt which would set off the decomposition reaction leading to the production of nanoparticles (Fig. 8—from 220 °C).

**Fig. 9:**
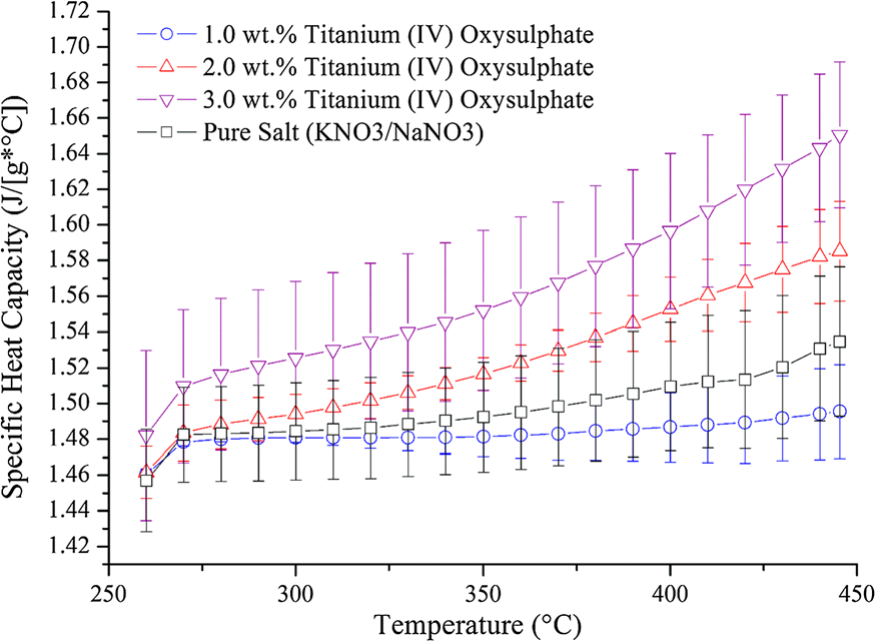
Specific heat capacity of TiO_2_ nanoparticles produced in situ compared to pure nitrate salt (60 wt% NaNO_3_/40 wt% KNO_3_)

The production of TiO_2_ within molten salt phase seemed to provide on average an enhancement which was dependent on the amount of precursor utilize in the synthesise process, although the latter cannot be deemed reliable for 1.0 and 2.0 wt% of TiOSO_4_ due to the overlapping error bars. Still a trend is seen. At a concentration of 3.0 wt% of TiOSO_4_, a significant increase is obtained above 390 °C with a 5.4 % increase compared to base salt, rising to 7.5 % at 445 °C (Fig. 9). Providing a way to disperse the aggregate produced during the formation of TiO_2_ might enhance the specific heat capacity through the production of a more homogenous mix.

Clearly, the production in situ of nanoparticles does alter the storage capacity of molten salt which still raise the questions as to the exact mechanisms allowing these anomalous enhancements to occur as nanoparticles themselves have lower specific heat capacity compared to that of the salt used. Various hypotheses are mentioned in the literatures:

- Nano-layers—Occurring at the interface of the liquid salt and the solid nanoparticles, the molten salt ions would be constrained into a semi-solid phase causing the production of layers with increase degree of freedoms away from the interface. The partial incorporation of the enthalpy of fusion in this semi-solid structure could partly provide an explanation for the rise in Cp (Jo and Banerjee 2014). This was observed by Oh et al. (2005) research team with the layering of aluminium atoms at the interface of sapphire (Oh et al. 2005). We hypothesise that these adsorbed layers of fluid or solvent molecules with nano-scale thickness would be strongly affected by the shape of the nanoparticles.
- Interfacial Thermal or Kapitza Resistance—Storage at the interface of the solid and liquid due to the differences in electronic and vibrational properties of the different materials. This could be enhanced by the large surface area-to-volume ratio of nanoparticles (Shin and Banerjee 2011a, b).
- Higher Cp of nanoparticles compared to their bulk counterpart. Reported by Tan et al. (2009), the increased exposure of surface atoms is showed to increase the Cp value measured in various tested nanoparticles (Tan et al. 2009). This mode is unlikely to play a major role in enhancement but might contribute to it.

Another hypothesis could also be:

- Entrapment of molten salt in nanoparticle agglomerates could also promote the enhancement of Cp through the semi-solid arrangement of ions (Shin and Banerjee 2011a, b; Jo and Banerjee 2015). However, as seen in the EDX map, the wetting behaviour is crucial to the interaction between the nanoparticles and the molten salt.

Whilst each of these modes might play a certain extent in the enhancement of Cp, adsorbed layer of nano-scale thickness and entrapment of molten salt in agglomerate are likely to be the major component of this rise. What is clear is that the development of in situ production and dispersion of nanoparticles in molten salt phase would pave the way toward a cost-effective alternative toward the enhancement of specific heat capacity were it successful. However, the problem of agglomeration of nanoparticle is still a variable that need to be looked into for future research. Furthermore, this method could also be used in the production of nanoparticles, although separating the nanoparticle from the salt would be challenging.

## Conclusions

In this study, the production of TiO_2_ nanoparticles was successfully achieved in a molten salt phase through the use of inexpensive precursors (TiOSO_4_). The produced nanoparticles were relatively homogenous in size (16.35 nm), although highly agglomerated. EDX mapping revealed that potassium ions could pack more tightly to the titanium oxide nanoparticle than sodium. DSC analysis showed that the increase in precursors was only significant when using 3.0 wt% of TiOSO_4_ precursors and at temperature of 390 °C and above.

Future work would concentrate on defining how the ratio of precursor used would affect the nanoparticle size as well as the time and temperature utilized to produce them. Furthermore, defining ways to destroy the formed aggregate might also prove useful in enhancing the Cp value. This is likely to affect the thermo-physical properties of the salt and a more detailed characterisation of these changes would need to be carried out.
